# Supplementary material for: Impact of Anemia Severity on the Outcome of an Aneurysmal Subarachnoid Hemorrhage
Source: J Clin Med. 2022 Oct 24;11(21):6258. doi: 10.3390/jcm11216258 (PMC9657573; doi:10.3390/jcm11216258)
Supplement: Supplementary file 1 [file jcm-11-06258-s001.zip › jcm-1954503-supplementary.pdf]

SUPPLEMENTARY MATERIALS

Supplementary Table S1: Univariate analysis on the association between different nHb values and potential prognostic factors.

Variable \ Nadir Hb	<7.0 g/dL		<8.0 g/dL		<9.0 g/dL		<10.0 g/dL		<11.0 g/dL	
	OR (95% CI)	p-value	OR (95% CI)	p-value	OR (95% CI)	p-value	OR (95% CI)	p-value	OR (95% CI)	p-value
Demographic parameters and comorbidities										
Age ≥55 years	1.95 (0.90-4.19)	0.09	**1.81 (1.18-2.79)**	**0.007**	**1.87 (1.36-2.57)**	**<0.0001**	**1.42 (1.01-1.98)**	**0.042**	1.36 (0.89-2.07)	0.168
Female sex	0.85 (0.40-1.84)	0.692	0.86 (0.56-1.34)	0.498	1.11 (0.80-1.55)	0.554	**1.54 (1.09-2.17)**	**0.016**	**2.36 (1.55-3.60)**	**<0.0001**
Ethnicity (non-Caucasian)	0.77 (0.10-5.89)	1.000	1.47 (0.58-3.71)	0.427	1.27 (0.60-2.72)	0.559	1.79 (0.72-4.49)	0.301	1.71 (0.51-5.76)	0.603
Hypertension	1.59 (0.64-3.98)	0.404	**1.82 (1.08-3.07)**	**0.024**	**1.45 (1.02-2.07)**	**0.042**	1.33 (0.93-1.91)	0.135	1.07 (0.68-1.69)	0.815
Hypercholesterolemia	1.27 (0.37-4.36)	0.727	1.59 (0.80-3.14)	0.176	1.34 (0.76-2.36)	0.310	**2.51 (1.20-5.24)**	**0.013**	2.03 (0.79-5.23)	0.178
Hypothyroidism	1.59 (0.59-4.29)	0.398	1.10 (0.58-2.09)	1.000	1.06 (0.66-1.73)	0.904	1.62 (0.93-2.83)	0.205	1.37 (0.68-2.76)	1.000
Hyperthyroidism	1.87 (0.10-34.63)	1.000	0.47 (0.03-8.63)	1.000	2.19 (0.36-13.21)	0.403	1.92 (0.21-17.29)	1.000	2.24 (0.12-40.79)	0.596
Hyperuricemia	2.71 (0.59-12.40)	0.198	2.05 (0.71-5.87)	0.190	1.87 (0.73-4.79)	0.226	1.26 (0.44-3.58)	0.801	1.64 (0.37-7.22)	0.751
Diabetes mellitus	1.18 (0.27-5.16)	0.689	1.20 (0.51-2.81)	0.649	1.49 (0.77-2.87)	0.238	**4.31 (1.51-12.33)**	**0.002**	**7.85 (1.07-57.84)**	**0.012**
Renal diseases	0.38 (0.02-6.47)	0.622	1.32 (0.48-3.58)	0.580	0.98 (0.43-2.21)	1.000	0.85 (0.37-1.96)	0.670	0.80 (0.29-2.18)	0.592
Regular medication										
Statins	1.38 (0.31-6.05)	0.658	1.68 (0.74-3.80)	0.228	1.24 (0.63-2.47)	0.596	**2.99 (1.14-7.83)**	**0.024**	2.17 (0.65-7.22)	0.246
Painkiller abuse	0.43 (0.06-3.24)	0.715	0.60 (0.23-1.57)	0.408	1.0 (0.55-1.83)	1.000	0.63 (0.34-1.15)	0.145	0.73 (0.35-1.52)	0.417
Blood thinners	2.28 (0.84-6.24)	0.166	1.49 (0.76-2.94)	0.252	1.39 (0.80-2.41)	0.257	1.84 (0.95-3.57)	0.073	1.75 (0.73-4.18)	0.262
Initial SAH severity										
WFNS grade (4-5)	2.14 (0.99-4.60)	0.056	**2.85 (1.83-4.45)**	**<0.0001**	**3.03 (2.19-4.21)**	**<0.0001**	**3.18 (2.21-4.57)**	**<0.0001**	**3.27 (2.01-5.31)**	**<0.0001**
Fisher grade (3-4)	7.91 (0.48-131.0)	0.063	**4.76 (1.46-15.46)**	**0.003**	**2.58 (1.44-4.64)**	**<0.0001**	**1.87 (1.12-3.15)**	**0.024**	**2.14 (1.17-3.91)**	**0.018**
Acute hydrocephalus	2.0 (0.68-5.83)	0.265	**3.30 (1.67-6.51)**	**<0.0001**	**3.02 (1.98-4.60)**	**<0.0001**	**2.83 (1.94-4.12)**	**<0.0001**	**2.75 (1.77-4.26)**	**<0.0001**
Aneurysm size ≥7mm	1.54 (0.72-3.32)	0.333	1.22 (0.80-1.87)	0.383	1.15 (0.83-1.58)	0.417	1.14 (0.81-1.59)	0.496	1.24 (0.81-1.89)	0.338

Clinical events and complications during SAH										
Aneurysm rebleed	2.3 (0.66-8.04)	0.174	1.54 (0.65-3.66)	0.321	1.31 (0.64-2.67)	0.465	2.15 (0.87-5.30)	0.120	1.99 (0.59-6.65)	0.335
Vasospasm	1.63 (0.75-3.51)	0.210	1.44 (0.92-2.27)	0.118	**1.74 (1.23-2.47)**	**0.002**	**2.78 (1.82-4.26)**	**<0.0001**	**3.58 (1.91-6.71)**	**<0.0001**
DC	1.64 (0.77-3.50)	0.218	**2.71 (1.76-4.18)**	**<0.0001**	**3.09 (2.18-4.37)**	**<0.0001**	**5.32 (3.31-8.55)**	**<0.0001**	**7.85 (3.57-17.24)**	**<0.0001**
ACS	1.36 (0.17-10.7)	0.543	**3.0 (1.08-8.35)**	**0.039**	2.14 (0.80-5.71)	0.137	2.33 (0.66-8.20)	0.202	1.46 (0.33-6.49)	1.000
Systemic infection	0.91 (0.42-1.97)	0.848	**1.71 (1.11-2.63)**	**0.02**	**2.28 (1.63-3.18)**	**<0.0001**	**3.23 (2.20-4.75)**	**<0.0001**	**3.92 (2.27-6.77)**	**<0.0001**
Clipping	0.64 (0.29-1.42)	0.335	**1.79 (1.17-2.73)**	**0.008**	**2.44 (1.76-3.37)**	**<0.0001**	**4.22 (2.86-6.24)**	**<0.0001**	**6.99 (3.75-13.05)**	**<0.0001**

Abbreviations: Hb=hemoglobin, OR= odds ratio, CI= confidence interval, WFNS= World Federation of Neurosurgical Societies, DC= decompressive craniectomy, ACS= acute coronary syndrome. Significant results are in bold.

Supplementary Table S2: Multivariate analysis with the M1 (adjusted for baseline characteristics) and M2 models on the association between different nHb values and the major outcome endpoints of the study.

**Nadir Hb:**		**<7.0 g/dL**		**<8.0 g/dL**		**<9.0 g/dL**		**<10.0 g/dL**		**<11.0 g/dL**	
		OR (95% CI)	p-value	OR (95% CI)	p-value	OR (95% CI)	p-value	OR (95% CI)	p-value	OR (95% CI)	p-value
Cerebral infarctions	MVA M1	1.29 (0.58 - 2.87)	0.530	1.54 (0.96 - 2.46)	0.073	**1.72 (1.21 - 2.46)**	**0.003**	**2.47 (1.66 - 3.68)**	**<0.0001**	**2.30 (1.38 - 3.84)**	**0.001**
	MVA M2	1.28 (0.56 - 2.96)	0.557	1.33 (0.82 - 2.17)	0.249	**1.70 (1.16 - 2.50)**	**0.007**	**1.83 (1.19 - 2.81)**	**0.006**	1.67 (0.98 - 2.86)	0.062
Unfavorable outcome at 6 months	MVA M1	1.98 (0.80 - 4.92)	0.142	**2.85 (1.65 - 4.92)**	**<0.0001**	**3.05 (2.04 - 4.56)**	**<0.0001**	**2.19 (1.39 - 3.46)**	**0.001**	**2.17 (1.18 - 3.98)**	**0.013**
	MVA M2	1.98 (0.74 - 5.34)	0.176	**2.21 (1.21 - 4.04)**	**0.010**	**2.44 (1.57 - 3.79)**	**<0.0001**	1.50 (0.89 - 2.52)	0.126	1.43 (0.75 - 2.72)	0.280
In-hospital mortality	MVA M1	2.43 (0.64 - 9.21)	0.193	**3.32 (1.62 - 7.23)**	**0.003**	**4.84 (2.49 - 9.41)**	**<0.0001**	**9.61 (4.08 - 22.62)**	**<0.0001**	**7.41 (2.74 - 20.04)**	**<0.0001**
	MVA M2	2.20 (0.50 - 9.68)	0.298	**2.44 (1.07 - 5.55)**	**0.034**	**3.22 (1.59 - 6.51)**	**0.001**	**6.06 (2.39 - 15.34)**	**<0.0001**	**4.60 (1.61 - 13.14)**	**0.004**

Abbreviations: OR= odds ratio, CI= confidence interval. Significant findings are in bold.

Supplementary Table S3: Analysis of the association between the timing of outcome-relevant nHb <9.0 g/dL and the major study endpoints.

**nHb < 9.0 g/dL**		**Begin**			**Duration**		
		Median (days)	95% CI	p-value	Mean (days)	SD	p-value
Cerebral infarctions	yes	6	4.58-7.00	0.504	4	4	0.488
	no	5	4.00-6.67		5	5	
Unfavourable outcome at 6 months	yes	5	4.00-6.00	0.382	4	5	0.662
	no	5.4	4.00-7.00		4	5	
In-hospital mortality	yes	**3.9**	**2.23-5.00**	**0.001**	**3**	**3**	**0.041**
	no	**6**	**5.67-7.60**		**5**	**5**	

Abbreviations: CI = confidence interval, SD = standard deviation. Significant findings are bold.
